# Epigenetic inflammatory memory and periodontal disease: Mechanisms and clinical significance for comorbidities

**DOI:** 10.1002/jper.70040

**Published:** 2025-12-26

**Authors:** George Hajishengallis

**Affiliations:** ^1^ Department of Basic and Translational Sciences Penn Dental Medicine, University of Pennsylvania Philadelphia Pennsylvania USA

**Keywords:** clonal hematopoiesis, comorbidities, inflammation, periodontitis, trained immunity

## Abstract

Historically, immunological memory was considered an exclusive feature of adaptive immunity. However, innate immune cells have recently been shown to record and maintain epigenetically imprinted memory of earlier infectious or inflammatory challenges. In the bone marrow, hematopoietic stem and progenitor cells (HSPCs) can also build inflammatory memory, which leads to trained myelopoiesis, that is, the production of high numbers of myeloid cells with increased inflammatory responsiveness to future challenges. Another condition affecting HSPCs and causing overproduction of leukocytes with increased inflammatory responsiveness is clonal hematopoiesis of indeterminate potential (CHIP). Occurring at high prevalence in the elderly, CHIP is driven predominantly by somatic mutations in genes encoding epigenetic modifiers, hence altering the epigenetic landscape of hematopoietic progenitors and their mature progeny. Although trained myelopoiesis and CHIP may have beneficial effects, they can also act in a maladaptive context and aggravate inflammation in periodontal disease and systemic conditions, thereby forming a common mechanistic basis for inflammatory comorbidities. This review discusses recent experimental and clinical evidence on the mechanisms and implications of maladaptive hematopoiesis—due to trained myelopoiesis or CHIP—‐in periodontitis and associated inflammatory comorbidities.

**Plain language summary:**

Traditionally, immune memory—the ability to “remember” past infections—was thought to be limited to the adaptive immune system. But recent discoveries show that even the more ancient part of the immune system, the innate immune system, can also develop a form of memory. This memory is stored through changes in how genes are regulated, and it begins in the bone marrow. There, blood‐forming stem cells can be “trained” by previous infection or inflammation to produce more immune cells that respond more aggressively to future threats, a process known as trained myelopoiesis. An analogous process happens in a condition called clonal hematopoiesis of indeterminate potential (CHIP), which is common in older adults. In CHIP, mutations in certain genes cause the bone marrow to overproduce immune cells that are overly reactive. While trained myelopoiesis and CHIP may exert protective effects, they can also backfire. Both trained myelopoiesis and CHIP have been linked to increased inflammation in gum disease and associated systemic conditions. This review explores how bone marrow–driven changes in the production and activity of immune cells may contribute to a shared underlying cause of multiple inflammatory disorders and why understanding these processes could open new doors for treatment and prevention.

## 1 INTRODUCTION

Periodontal disease has a complex etiology that acts at different levels. At the level of the host, factors such as genetics, age, sex and systemic health status may predispose to or protect from periodontitis.^1^, ^2^ At the level of the microbiome, the microbial species composition influences the interactions of the periodontal microbial community with the host towards a dysbiotic or eubiotic relationship; the reverse is also true, as the host response influences the microbiome.^3^ Etiological factors of periodontitis also act at the level of the exposome, that is, the sum of environmental and lifestyle factors (e.g., diet, stress, physical activity, and, smoking) we are exposed to and which interact with our individual characteristics to influence our health.^4^, ^5^ Such factors contribute to epigenetic changes that influence the expression of host response genes and hence may promote resistance or susceptibility to periodontitis.

Periodontitis remains a serious public health and economic burden and is linked with increased risk of systemic chronic conditions (comorbidities), including atherosclerosis, rheumatoid arthritis, type‐2 diabetes, Alzheimer's disease, and metabolic dysfunction‐associated steatotic liver disease.^6^, ^7^, ^8^ Although periodontal disease shares genetic and environmental risk factors with the aforementioned systemic disorders, an independent association between periodontitis and comorbidities persists even following adjustment for confounders, such as age, smoking, and obesity.^9^, ^10^, ^11^, ^12^ Periodontitis and other chronic inflammatory diseases become more prevalent with aging and are associated with “inflammaging”; the latter represents the aging‐related elevation of low‐grade chronic systemic inflammation, typified by increased concentrations of interleukin (IL)‐6, tumor necrosis factor, and IL‐1.^13^, ^14^, ^15^, ^16^ Periodontitis can contribute to inflammaging through the spillover of locally produced inflammatory mediators into the bloodstream or through transient bacteremias and consequent inflammatory responses in the circulation.^8^, ^17^, ^18^ Compared to healthy individuals, periodontitis patients have increased concentrations of proinflammatory mediators (e.g., IL‐1, IL‐6, C‐reactive protein) and neutrophil counts in the blood circulation.^8^, ^19^, ^20^ Therefore, periodontitis can instigate low‐grade systemic inflammation and thereby influence comorbidities. The relationship between periodontal disease and systemic comorbidities is bidirectional. In this regard, systemic inflammatory disorders can increase the inflammatory burden on the periodontium and thereby exacerbate periodontitis.^12^, ^21^, ^22^

A number of mechanisms have been proposed to underlie the comorbid connection of periodontitis and systemic disorders, some of which are adequately supported and have been described in detail elsewhere.^8^, ^22^ For instance, there is convincing evidence that periodontal pathogens promote the generation of anti‐citrullinated protein antibodies that contribute to the pathogenesis of rheumatoid arthritis.^23^, ^24^, ^25^ However, it was only recently that unifying causal mechanisms have been proposed to explain the bidirectional association of periodontitis with comorbidities and the fact that susceptibility to these disorders increases with aging. These newly emerged mechanisms, namely trained immunity (TRIM) and clonal hematopoiesis of indeterminate potential (CHIP), are the focus of this review. As discussed in detail below, both mechanisms influence the epigenetic landscape and memory of innate immune cells in ways that create persistent hyper‐responsive phenotypes that contribute to immune‐mediated pathology. TRIM can be induced in mature myeloid cells in the periphery (peripheral TRIM) or in long‐lived hematopoietic stem and progenitor cells (HSPCs) in the bone marrow (central TRIM or trained myelopoiesis).^26^ Whereas central TRIM is based on sustained, yet potentially reversible, inflammatory epigenetic memory, the epigenetic changes associated with CHIP are fixed, thus in essence representing a permanent memory state.^27^ Experimental and clinical evidence will be presented in support of the concept that central TRIM and CHIP can each contribute to the association between periodontal and systemic inflammatory diseases.

## 2 CENTRAL TRIM AND CHIP AND THEIR IMPACT ON CHRONIC INFLAMMATION

**FIGURE 1:**
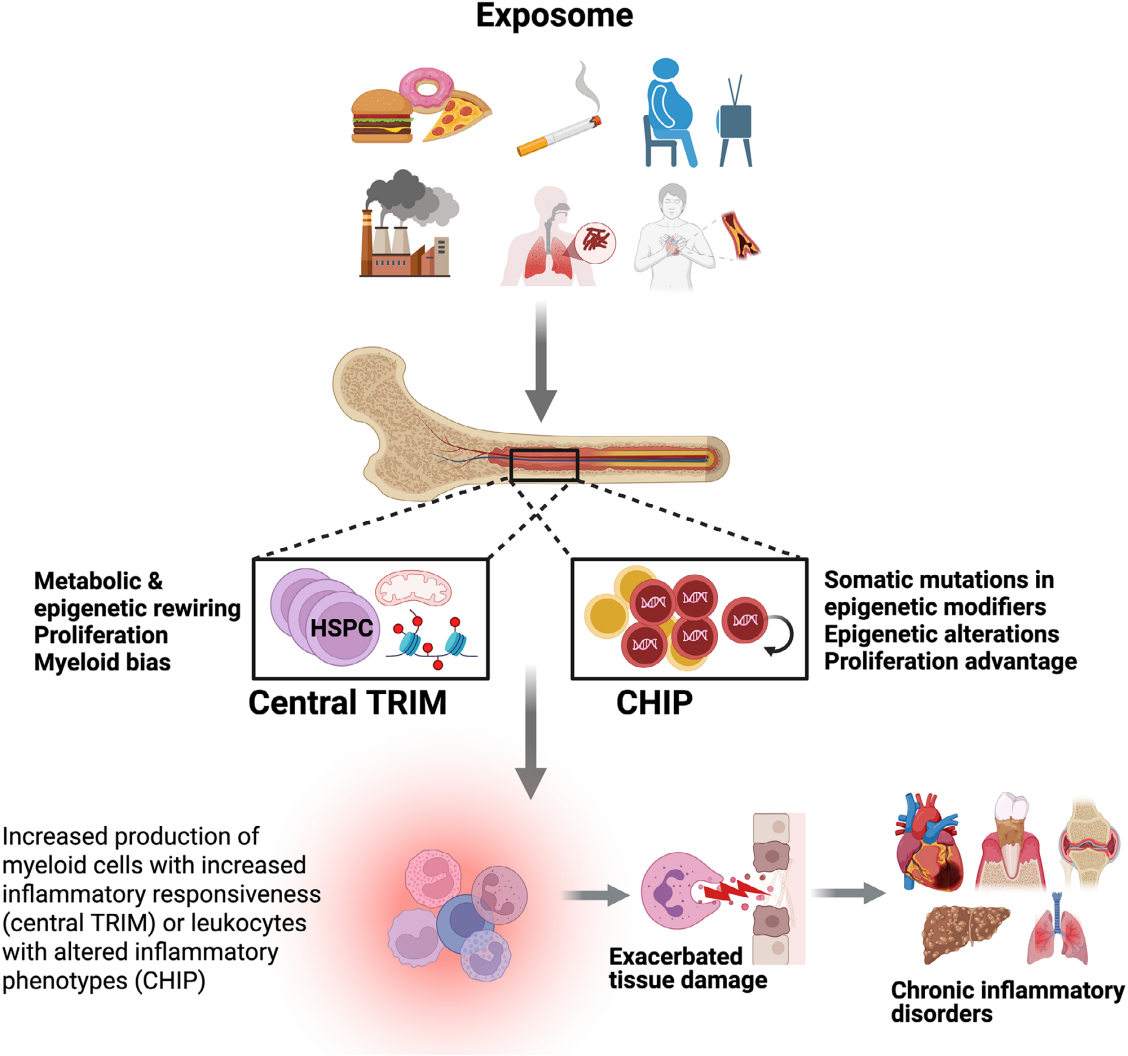
Exposome‐driven predisposition to maladaptive trained immunity (TRIM) and clonal hematopoiesis of indeterminate potential (CHIP). Environmental and lifestyle factors such as environmental pollutants/toxins, unhealthy diet, physical inactivity/obesity, smoking, and chronic infections or inflammatory diseases promote the induction of central TRIM and the emergence of CHIP, thereby promoting the generation of hyper‐inflammatory immune cells and facilitating the exacerbation of periodontitis and other chronic inflammatory conditions.

Given that chronic diseases are largely driven by the action of inflammatory immune cells, epigenetic alterations in their bone marrow precursors, the HSPCs, may affect multiple disorders that emerge as comorbidities. Alterations to HSPCs giving rise to progeny with elevated proinflammatory potential may result from two non‐mutually exclusive phenomena, central TRIM and CHIP. A number of environmental and lifestyle factors that are known to influence periodontitis are also factors that promote central TRIM and CHIP. Thus, unhealthy diet, physical inactivity/obesity, smoking, as well as chronic infections or inflammatory diseases facilitate the induction of central TRIM ^28^, ^29^, ^30^, ^31^ and the emergence of CHIP ^32^, ^33^, ^34^, ^35^, ^36^ (Figure 1). Germline genetic variation may also predispose to CHIP. Several inherited polymorphic loci have been identified that influence CHIP risk. For example, a common deletion (rs34002450) at the telomerase reverse transcriptase (TERT) locus and germline variants in Runt‐related transcription factor 1 (RUNX1), a key transcription factor in hematopoiesis, are associated with increased predisposition to CHIP.^37^, ^38^, ^39^, ^40^ Interestingly, not all germline variants confer increased risk. Two missense variants in *LY75*, a gene encoding a lymphocytic antigen, are linked to reduced incidence of *DNMT3A*‐driven CHIP. Additionally, a common polymorphism (rs2887399) in the promoter region of the T cell lymphoma/leukemia 1A gene (*TCL1A*) is associated with attenuated expansion of hematopoietic stem cell (HSC) clones harboring mutations in *TET2* and *ASXL1*, but not in *DNMT3A*. Host genetic variability such as genetic polymorphisms in *IL1B* influence the induction of TRIM by the bacillus Calmette–Guérin (BCG) vaccine.^41^, ^42^ Despite these insights, the broader relationship between germline variants and TRIM remains underexplored. It is plausible that genetic differences in pathways regulating glycolysis and histone modification may affect the ability of hematopoietic progenitors and mature myeloid cells to undergo innate immune training, warranting further investigation.

### 2.1 Inflammatory memory and the trained immune response

The bone marrow represents the epicenter of hematopoietic cell production, where HSPCs produce all types of blood cells.^43^ By expressing receptors for microbial products and for cytokines, HSPCs can detect systemic infection or inflammation that affects the bone marrow. In response to such infectious and/or inflammatory challenges, HSPCs proliferate and initiate elevated production of myeloid cells (e.g., neutrophils and monocytes) to swiftly replenish those consumed in the periphery.^44^ This acute demand‐driven hematopoietic process of heightened de novo generation of myeloid cells is known as emergency myelopoiesis.^44^

Under inflammatory conditions, HSPCs may become adapted for long‐standing augmented proliferation and biased differentiation toward the myeloid‐cell lineage. This inflammatory adaptation of HSPC is primarily driven by epigenetic changes that also underlie the induction and maintenance of central TRIM.^44^, ^45^, ^46^, ^47^ Such epigenetic adaptations can be largely retained even after the inflammatory stimulus has subsided, thereby forming the basis for a sustained and recallable innate immune (or inflammatory) memory. It is important to clarify that emergency myelopoiesis is an acute process that does not necessarily lead to central TRIM, which is a long‐term state.^48^ Although during its induction, central TRIM shares features of emergency myelopoiesis,^46^ the latter might ultimately inflict severe stress to HSCs, resulting in their exhaustion, rather than leading to innate immune training.^26^, ^49^, ^50^

Once an epigenetic state of innate immune readiness (innate immune memory) has been established, the chromatin—although transcriptionally inert—remains readily accessible and is thus conducive to quicker recruitment of transcription factors, leading to augmented transcription of target genes upon future stimulation.^51^ Specifically, the persistent epigenetic changes in inflammation‐trained HSPCs enhance the accessibility of enhancers and promoters involved in myeloid‐lineage differentiation and inflammatory responses, consequently leading to elevated transcription of the respective genes upon a future challenge.^31^, ^47^ This epigenetic rewiring underlies the sustained enhancement of myelopoiesis —termed trained myelopoiesis—which leads to the generation of myeloid cells that can release increased levels of inflammatory factors in response to microbial or inflammatory stimuli. It is implicit from the above that epigenetic adaptations in hematopoietic progenitors have functional consequences in progeny myeloid cells. This is because epigenetic changes in progenitor cells can be inherited downstream during cell differentiation.^51^, ^52^, ^53^ In other words, hematopoietic progenitors preserve inflammation‐induced epigenetic signatures and transmit these signatures to myeloid progeny, thereby sustaining a circulating and recruitable pool of innate immune cells with inflammatory memory. Trained HSPCs can thus contribute to perpetuation of inflammation (i.e., shifting an acute response to chronic) both quantitatively, by elevated and sustained production of myeloid cells, and qualitatively, by generation of myeloid cells with increased inflammatory responsiveness.^44^, ^54^, ^55^

A distinct component of myeloid biology, osteoclastogenesis, can also undergo innate immune training by inflammatory stimuli. The training involves epigenetic alterations in monocyte/macrophage‐lineage precursors of osteoclasts, which thus acquire increased predisposition toward the generation of mature osteoclasts, thereby leading to exacerbated inflammatory bone loss.^56^

Although innate immune training represents an evolutionarily conserved mechanism that promotes host survival upon re‐infection,^41^, ^57^, ^58^ it may also promote maladaptive inflammatory responses that exacerbate chronic inflammatory diseases, including bone loss disorders,^26^, ^28^, ^31^, ^59^, ^60^ as discussed in Section 3.

### 2.2 Clonal hematopoiesis, a fixed state of maladaptive hematopoiesis

**FIGURE 2:**
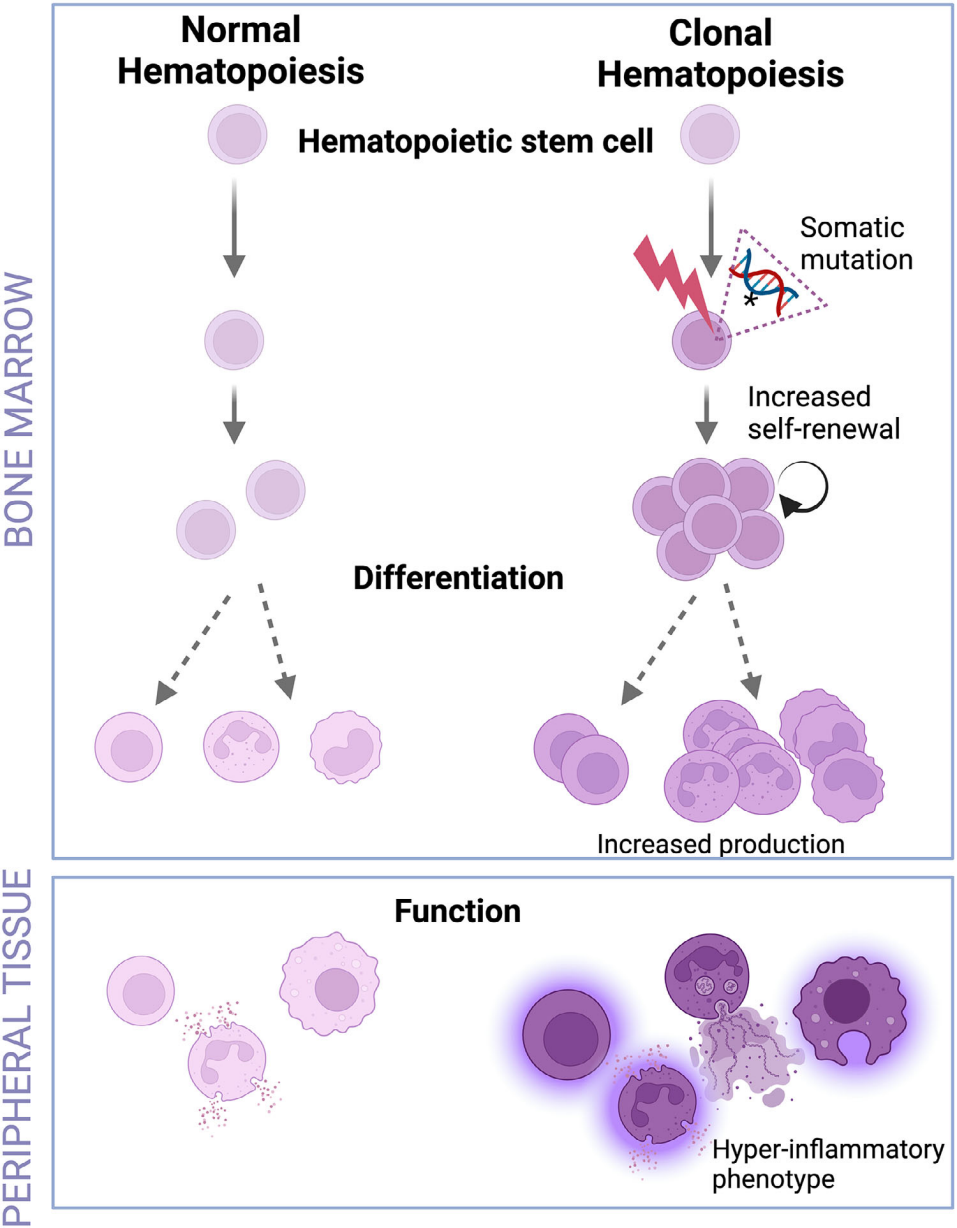
Clonal hematopoiesis of indeterminate potential (CHIP) and altered leukocyte phenotypes. With advancing age, hematopoietic stem cells may acquire somatic mutations, some of which endow the cell with increased self‐renewal capacity, relative to normal stem cells. The increased self‐renewal and expansion of CHIP‐mutant hematopoietic stem cells leads to the production of an outsized fraction of mutant leukocytes (relative to those produced during normal hematopoiesis), which typically have increased inflammatory responsiveness and can aggravate inflammation in peripheral tissues.

With increasing age, somatic mutations accumulate in HSPCs, some of which confer a competitive advantage to the mutant HSPC for increased proliferation relative to a wild‐type HSPC. Such mutations therefore lead to increased expansion of the mutant HSPC clone and the generation of an outsized fraction of mutant progeny leukocytes, which typically have a hyper‐inflammatory phenotype (Figure 2). In the absence of evident hematological malignancy or cytopenia, this aging‐related expansion of blood cell clones harboring a somatic mutation is designated CHIP.^27^, ^61^ The “indeterminate potential” component of the term denotes the uncertainty of how CHIP may develop clinically, because often environmental stress factors are required to cooperate with the underlying somatic mutation to induce the disease phenotype.^44^, ^62^, ^63^, ^64^ CHIP‐mutant clones are infrequent in young adults but increase later in life, affecting more than 10% of individuals older than 65 years ^37^, ^65^, ^66^. These estimates are based on a variant allele fraction (VAF) threshold set at 2%; therefore, by definition, CHIP mutations are detectable at a VAF of 2% or greater. Besides its dependence on aging, the emergence of CHIP can be driven also by selective pressure from smoking or cytotoxic chemotherapy.^67^

As alluded to above, individuals with CHIP do not bear clinical signs of a hematologic disorder, such as myeloid malignancies or cytopenias.^65^, ^68^ Nevertheless, CHIP mutation carriers have an increased risk of developing hematologic malignant disorders, as well as atherosclerosis, diabetes mellitus and inflammation‐related disorders, such as chronic liver or kidney disease and periodontitis.^65^, ^66^, ^69^, ^70^, ^71^, ^72^, ^73^, ^74^, ^75^ Despite its association with disease states, CHIP may have beneficial effects, such as enabling longstanding hematopoiesis in the elderly, in which normal hematopoietic stem cells may be exhausted and thus CHIP clones may compensate for this failure.^76^, ^77^ It has also been reported that CHIP is associated with reduced risk of Alzheimer's disease; although Mendelian randomization analysis suggested that this may be a causal connection, the mechanisms involved are poorly understood.^78^

CHIP mutations typically cause loss of function and often affect three genes that encode epigenetic modifiers, namely, the enzymes *DNMT3A* (*DNA methyltransferase 3A*), the most frequently mutated gene in CHIP, *TET2* (*Ten‐eleven translocation methylcytosine dioxygenase 2*) and *ASXL1* (*Associated sex combs‐like 1*). *DNMT3A* and *TET2* catalyze cytosine methylation and demethylation, respectively, and mutations in these two genes jointly represent roughly 65% of all known CHIP mutations.^37^, ^65^, ^66^, ^68^, ^79^ The elevated inflammatory responsiveness typically seen in CHIP‐mutant myeloid cells may at least in part derive from epigenetic transcriptional priming, owing to the altered function of mutated epigenetic regulators, such as DNMT3A and TET2.^27^, ^80^, ^81^

## 3 CENTRAL TRIM AS A MECHANISTIC BASIS FOR INFLAMMATORY COMORBIDITIES

Although induction of central TRIM was shown to confer protection against impending challenges such as infections and cancer,^52^, ^82^, ^83^ central TRIM may also act in a maladaptive manner, thereby leading to persistent and amplified myeloid cell responses that potentially exacerbate distinct chronic inflammatory conditions.^6^ Here it should be clarified that “maladaptive” TRIM is not intrinsically dysfunctional. TRIM can amplify, rather than initiate, the inflammatory response and whether the outcome of the amplified response is protective or maladaptive depends on the specific context in which TRIM acts, such as the health status, genetic background, the exposome (e.g., diet and lifestyle) or the age of the host.^26^

**FIGURE 3:**
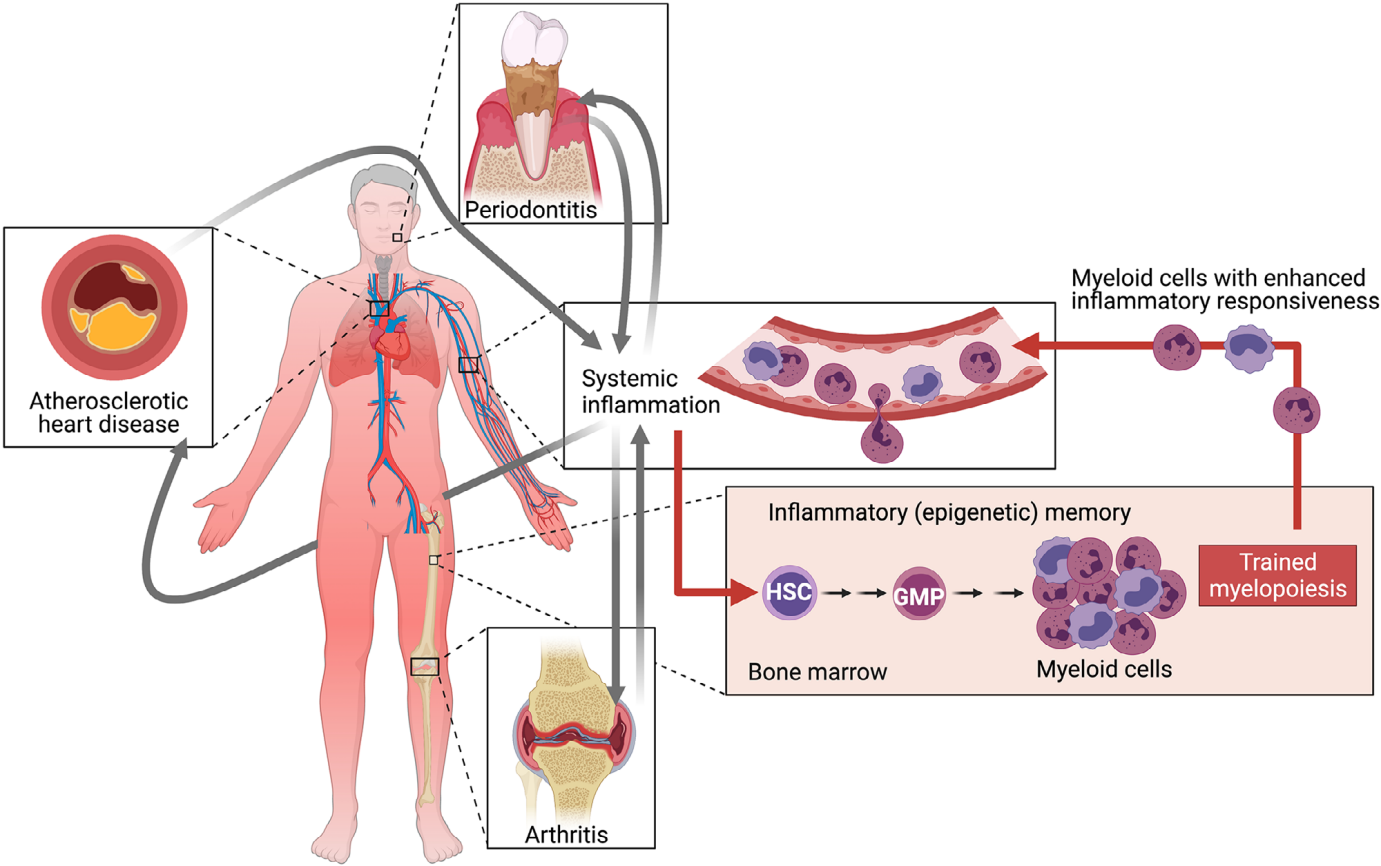
Inflammatory memory in bone marrow hematopoietic progenitors underlies the pathogenesis of inflammatory comorbidities. Systemic inflammation associated with inflammatory diseases is sensed in the bone marrow by hematopoietic stem and progenitor cells, which are epigenetically rewired and undergo myeloid‐biased differentiation, thus producing high numbers of myeloid cells with increased inflammatory responsiveness (trained myelopoiesis). These hyper‐responsive myeloid cells circulate and populate sites of infection or inflammation, such as the periodontal tissue, the joints and the arterial wall. They can thus amplify inflammation and exacerbate distinct inflammatory diseases that appear as comorbidities. For instance, periodontitis patients—who display signs consistent with inflammatory memory (see text)—have elevated risk of developing atherosclerosis and rheumatoid arthritis. GMP, granulocyte‐monocyte progenitor; HSC, hematopoietic stem cell. Adapted from Ref. 84 with permission.

The novel concept that TRIM in the bone marrow may mechanistically link inflammatory comorbidities has been experimentally established in the setting of bone loss disorders, namely periodontitis and rheumatoid arthritis.^31^ Specifically, it was shown that long‐term experimental periodontitis in mice (using the ligature‐induced periodontitis model) causes systemic inflammation and leads to the induction of epigenetically imprinted inflammatory memory in bone marrow HSPCs, which in turn generate elevated numbers of hyper‐responsive (trained) myeloid cells that mediate increased susceptibility to experimental arthritis (using the collagen antibody‐induced arthritis [CAIA] model).^31^ Induction of this trained phenotype requires intact IL‐1 receptor (IL‐1R) signaling in HSPCs, which can thereby undergo myeloid‐skewed differentiation and mediate trained myelopoiesis. This maladaptively trained phenotype can be transferred by transplantation of bone marrow from periodontitis‐experienced donor mice (which are periodontally healthy at the time of transplantation) to naive recipients. The recipient mice exhibit heightened inflammation and pathology in the joints when subjected to CAIA, as compared to controls, that is, recipients of normal (i.e., untrained) bone marrow.^31^ Therefore, maladaptive training of HSPCs appears to be a mechanistic basis that can link distinct inflammatory comorbidities (Figure 3).

In addition to external inflammatory and infectious stimuli, TRIM may also be instigated by host‐derived inflammatory molecules, such as oxidized low‐density lipoprotein and high glucose. For instance, in cardiometabolic disorders, oxidized low‐density lipoprotein can cause innate immune training of human monocytes in a manner dependent on IL‐1R signaling.^59^ Consistently, when fed a Western‐type diet to model metabolic syndrome‐induced inflammation, lipoprotein receptor‐deficient (*Ldlr^−/−^*) mice develop IL‐1R signaling‐dependent maladaptive central TRIM, which persists for at least 4 weeks after the mice are switched back to a normal (chow) diet.^59^ In these mice, epigenetic and transcriptomic rewiring in granulocyte–monocyte progenitors (GMPs) was associated with functional reprogramming of myeloid cells and increased systemic inflammation and atherosclerotic lesions.^59^ Hyperglycemia can also instigate enduring metabolic and epigenetic changes associated with TRIM in hematopoietic progenitors and mature myeloid cells, including macrophages.^85^ This hyperglycemia‐trained phenotype can be transferred via transplantation of bone marrow from streptozotocin‐induced diabetic mice to *Ldlr* ^−/−^ normoglycemic recipient mice. Importantly, these recipients—when fed a Western‐type diet—develop increased formation of atherosclerotic plaques as compared to controls that were transplanted with bone marrow from normoglycemic donors.^85^

Evidence for induction of TRIM—including innate immune training of hematopoietic progenitors associated with persistent elevated inflammatory responsiveness of circulating myeloid cells—is well documented in humans.^86^, ^87^ Clinical observations suggest a maladaptive trained immune phenotype associated with patients presenting with inflammatory or autoimmune diseases, including atherosclerosis, rheumatoid arthritis, periodontitis, and systemic lupus erythematosus ^6^, ^20^, ^88^. With regard to periodontitis in particular, multiple studies have shown that monocytes and neutrophils from the peripheral blood of patients release higher concentrations of proinflammatory cytokines following stimulation with microbial agonists ex vivo than similarly treated myeloid cells from periodontally healthy individuals; importantly, this immune hyper‐responsiveness could not be reversed even if the myeloid cells were isolated after successful periodontal therapy.^89^, ^90^, ^91^, ^92^, ^93^, ^94^, ^95^, ^96^ These findings of enduring inflammatory responsiveness in circulating innate immune cells suggest that periodontitis causes persisting inflammatory memory. The concept of maladaptive central TRIM can, in great part, explain why patients with periodontal disease may also present with arthritis and atherosclerosis, as well as other inflammatory comorbidities (Figure 3).

## 4 CHIP PROMOTES AND EXACERBATES INFLAMMATORY COMORBIDITIES

With the exception of myeloid malignancies, cardiovascular disease was the first pathological condition that was epidemiologically associated with CHIP mutations, such as in *DNMT3A* and *TET2*.^65^, ^66^ More recently, in a prospective study, CHIP driven by mutated *DNMT3A* or *TET2* was associated with elevated risk of de novo femoral atherosclerosis over a period of 6 years.^97^ The notion that the association of CHIP with atherosclerosis involves a cause‐and‐effect relationship was substantiated by experimental work in animal models. Transplantation of bone marrow cells with homozygous or heterozygous null mutations in the *Tet2* gene into *Ldlr* ^–/–^ mice resulted in aberrant expansion of the mutant clones and acceleration of atherosclerotic lesions as compared to *Ldlr* ^–/–^ control mice that received normal bone marrow cells.^69^, ^98^ Macrophages present in the atherosclerotic lesions of mice that received *Tet2* ^–/–^ bone marrow cells displayed higher production of IL‐1β (and other inflammatory cytokines) than *Ldlr* ^–/–^ controls receiving wild‐type bone marrow cells.^69^, ^98^ Accordingly, treatment of *Ldlr^−/−^* mice transplanted with *Tet2* ^–/–^ bone marrow cells with MCC950 or colchicine—both of which can inhibit the NLRP3 inflammasome and hence IL‐1β production ^99^, ^100^—caused significant attenuation of atherosclerotic plaque formation.^69^, ^101^ Consistent with these preclinical data, treatment with the anti‐IL‐1β monoclonal antibody canakinumab in patients with previous myocardial infarction (CANTOS trial) resulted in decreased incidence of major cardiovascular events, and notably of comorbidities associated with bone loss, namely arthritis, osteoarthritis, and gout.^102^ Consistently, patients with CHIP‐associated *TET2* mutations responded more favorably to the canakinumab treatment than patients with non‐TET2 mutant CHIP clones.^103^

CHIP has also been associated with increased risk of periodontitis and other chronic diseases (e.g., obstructive pulmonary disease, diabetes, chronic liver disease, chronic kidney disease, osteoporosis, rheumatoid arthritis, and gout).^65^, ^72^, ^74^, ^75^, ^76^, ^104^ Among 4946 community‐dwelling adults (ages 52‐74, mean age 62) of the dental component of the Atherosclerosis Risk in Communities (ARIC) study,^105^ 191 individuals (3.9% of total sample) were identified as CHIP carriers, the majority exhibiting mutations in *DNMT3A* (*n *= 118; 62% of total carriers) followed by *TET2* and *ASXL1*.^75^ Almost 60% of the CHIP carriers displayed VAF > 10%, ^75^ which is considered “large CHIP” and thus of greater risk of developing disease.^106^ The presence of CHIP‐associated *DNMT3A* mutations was significantly associated with severe periodontitis diagnosis (stage IV vs. stages I–III, as defined by the 2017 World Workshop Classification ^107^) and with the extent of interproximal clinical attachment loss and of gingival inflammation.^75^

Potential causality in this association between CHIP and periodontitis was tested in an in vivo model. Specifically, DNMT3A‐driven CHIP was modelled in mice with the heterozygous loss‐of‐function *Dnmt3a* mutation R878H, which is equivalent to the human hotspot mutation R882H in the *DNMT3A* gene. Partial transplantation with *Dnmt3a* ^R878H/+^ bone marrow cells (10% mutant cells, a clinically relevant VAF, and 90% wild‐type cells) resulted in preferential clonal expansion of mutant cells in recipient mice, which naturally developed periodontal inflammation and bone loss, in contrast to controls that received exclusively wild‐type bone marrow cells and remained periodontally healthy. Mice receiving *Dnmt3a* ^R878H/+^ bone marrow cells also became significantly more susceptible to the induction of experimental periodontitis and arthritis than controls receiving only wild‐type bone marrow cells. These heightened disease phenotypes, deriving from the presence of the *Dnmt3a* R878H mutation in hematopoietic cells, were associated with amplified osteoclastogenesis, IL‐17‐dependent and neutrophil‐mediated inflammation and defective regulatory T‐cell immunosuppressive activity.^75^ The transcriptomic and thus phenotypic changes in *Dnmt3a*‐mutant leukocytes are in great part due to DNA hypomethylation, in turn owing to defective methyltransferase function of mutated DNMT3A. DNA hypomethylation associated with the R878H mutation in HSPCs also resulted in upregulated expression of the mechanistic target of rapamycin (mTOR), which is known to prime quiescent stem cells for cell‐cycle entry.^75^, ^108^ It was thus reasoned that the mTOR inhibitor rapamycin could prevent DNMT3A‐driven CHIP. Indeed, treatment with rapamycin of mice receiving *Dnmt3a* ^R878H/+^ bone marrow cells was successful in blocking the aberrant clonal expansion of *Dnmt3a* ^R878H/+^ hematopoietic cells and consequently the development of periodontal inflammation and bone loss.^75^ Therefore, DNMT3A‐driven CHIP is a potentially treatable condition of maladaptive hematopoiesis that promotes inflammatory bone loss.

## 5 CONCLUSION AND PERSPECTIVE

Based on the above‐discussed recent literature, maladaptive TRIM acts as a common mechanistic basis for periodontitis and associated inflammatory comorbidities (such as arthritis and cardiovascular disease) (Figure 3) and CHIP can exacerbate all these inflammatory disorders (Figures 1 and 2). The maladaptive epigenetic alterations that drive trained myelopoiesis and increase susceptibility to periodontitis and inflammatory comorbidities can be long‐lasting. Trained immunity is thought to last for months, perhaps more than one year.^82^ Consistently, patients with successfully treated periodontitis retain augmented inflammatory responsiveness in peripheral blood myeloid cells, while the risk of cardiovascular disease does not necessarily subside during clinical remission of rheumatoid arthritis.^6^, ^96^ Moreover, in the context of hyperglycemia‐induced TRIM, glucose‐reducing treatments do not typically reduce the risk of cardiovascular complications in diabetic patients.^85^ Nevertheless, this epigenetically scripted inflammatory memory, which leads to maladaptive myelopoiesis, is in principle reversible. On the other hand, the altered epigenetic and transcriptomic state caused by CHIP mutations (in genes encoding epigenetic regulators such as DNMT3A and TET2) represents an essentially fixed state of maladaptive hematopoiesis. Thus, its adverse impact on aging‐related chronic inflammatory diseases, such as periodontitis, should be permanent unless there is specific intervention treatment.

In that regard, potential treatments (currently experimental) include blocking the aberrant expansion of CHIP‐mutant hematopoietic clones or restoring the defective activity of CHIP‐affected epigenetic enzymes (e.g., by enhancing the activity of the functionally intact enzyme expressed from the non‐mutant allele).^75^, ^108^, ^109^, ^110^, ^111^, ^112^ Given that CHIP is a prevalent condition affecting at least 10% of adults over 65 years and hence a significant public health issue,^68^, ^113^ screening for CHIP may identify individuals with increased risk for severe periodontitis and associated comorbidities. In the context of maladaptive TRIM, promising work in animal models and humans suggests the feasibility of developing clinical immunologic or epigenetic treatments to inhibit maladaptive TRIM and thus reduce the risk of multiple comorbidities.^88^, ^114^

The demonstration of an inflammatory periodontitis‐bone marrow axis in mice, that is, that periodontitis‐associated systemic inflammation causes trained myelopoiesis ^31^ is consistent with a clinical imaging study (18F‐fluorodeoxyglucose positron emission tomography/computed tomography), where the authors correlated periodontal metabolic activity (surrogate marker of inflammation) with hematopoietic tissue activity (reflecting stimulated myelopoiesis).^115^ However, formal evidence for periodontitis‐induced maladaptive trained myelopoiesis in humans is lacking. Such an investigation may be facilitated by studying hematopoietic stem cell transplant (HSCT) recipients. Thus, it would be particularly important to determine whether epigenetic inflammatory memory can be transmitted to HSCT recipients from donors with or without periodontal disease. The observation that inflammatory memory can be transmitted by bone marrow transplantation in mice ^31^ raises the possibility that the medical history (previous exposures to infectious and inflammatory challenges) of the donors may influence disease susceptibility in the recipients. This suggests that hematologists might need to consider inflammatory memory during the selection of appropriate donors for hematopoietic stem cell transplantations.

In conclusion, maladaptive innate immune training or CHIP‐associated epigenetic rewiring of HSPCs in the bone marrow constitute shared mechanisms for inflammatory comorbidities, including periodontal disease. This concept holds promise for the development of innovative therapeutic strategies targeting inflammatory comorbidities through a holistic approach, and merits further investigation.

## AUTHOR CONTRIBUTIONS

The author conceived, researched, and wrote this review article.

## CONFLICT OF INTEREST STATEMENT

The author declares no conflicts of interest.

## Exposome, TRIM, and CHIP: Interactions and therapeutic implications

The exposome encompasses the totality of environmental exposures an individual encounters throughout life, including factors such as diet, pollutants, infections, stress, smoking and other lifestyle behaviors. It complements the genome by accounting for non‐genetic influences on health and disease, and its dynamic interactions with the host genome and immune system are increasingly recognized as critical determinants of disease susceptibility and progression.^4^, ^5^ These factors may also modulate trained immunity (TRIM) and clonal hematopoiesis of indeterminate potential (CHIP), two processes rooted in hematopoietic stem and progenitor cells (HSPCs) that contribute to maladaptive immune responses. Exposomal inputs—such as microbial products, vaccines, and dietary components—can modulate TRIM by reprogramming HSPCs through metabolic shifts and epigenetic modifications (e.g., histone methylation/acetylation, DNA methylation).^116^, ^117^ These changes result in long‐term functional alterations in myeloid cell output, enhancing inflammatory responses upon secondary challenge. CHIP is influenced by stressors such as smoking, chemotherapy, radiation, unhealthy diet, and obesity. These exposures can accelerate clonal expansion and amplify the inflammatory phenotype of mutant clones.^33^, ^34^, ^35^, ^36^, ^118^ For example, smoking not only increases mutagenesis but also promotes the expansion of CHIP clones, thereby intensifying inflammatory risk ^36^, ^119^, ^120^. A key limitation in translating exposomal insights into therapeutic strategies is that while these exposures are associated with disease initiation and progression, their reversal may not necessarily restore immune homeostasis. This may be due to different reasons including the complexity of multiple interacting stressors, or the enduring effects of innate immune memory, wherein epigenetically imprinted immune hyper‐responsiveness may persist long after the exposomal trigger has been removed. To address these complexities, prospective longitudinal cohort studies are needed to track exposomal exposures and immune outcomes over time.^121^ Moreover, precision medicine approaches that integrate exposomal profiling with genomic and immunological data may guide individualized strategies to prevent or mitigate immune‐mediated diseases driven by maladaptive TRIM and/or CHIP.
